# Anatomical perspectives of brow‐lifting using threads: Clinical cases with techniques

**DOI:** 10.1111/jocd.16505

**Published:** 2024-08-12

**Authors:** Soo Yeon Park, Soo‐Bin Kim, Jovian Wan, Thanya Techapichetvanich, Ti Jo Tsay, Thanawan Sirisuk, Kyu‐Ho Yi

**Affiliations:** ^1^ Made‐Young Plastic Surgery Clinic Seoul Korea; ^2^ Division in Anatomy and Developmental Biology, Department of Oral Biology, Human Identification Research Institute, BK21 FOUR Project Yonsei University College of Dentistry Seoul Korea; ^3^ Asia Pacific Aesthetic Academy Hong Kong Hong Kong; ^4^ Department of Dermatology, Faculty of Medicine Siriraj Hospital Mahidol University Bangkok Thailand; ^5^ Ageless MD Clinic Tustin California USA; ^6^ Genitique Clinicm Bangkok Thailand; ^7^ Maylin Clinic (Apgujeong) Seoul Korea

**Keywords:** brow lifting, eyebrow thread lifting, rejuvenation, surgical techniques

## Abstract

**Introduction:**

Tailoring surgical interventions to target age‐related transformations is paramount. Many candidates seeking blepharoplasty commonly exhibit eyebrow ptosis, underscoring the necessity for surgeons to possess a comprehensive understanding of techniques for brow and forehead rejuvenation.

**Methods:**

Various surgical techniques are available for eyebrow and forehead enhancement, contrasting the standardized approaches in upper and lower blepharoplasty. Thread lifting has gained popularity for forehead lifting, although a more precise term would be eyebrow lifting. The thread lifting methods using V and I techniques with floating‐type threads (Secret Line, Hyundai Meditech., Inc., Wonju‐si, Republic of Korea) are used.

**Results:**

The natural aging process often causes the forehead and eyebrows to sag under the influence of gravity, leading to the appearance of heavy eyelids. Elevating the eyebrows can mitigate droopiness, enhance vision clarity, and rejuvenate the facial aesthetic. While non‐surgical methods like Botulinum Toxin A can weaken muscles and potentially aid in brow elevation, their visible effects may be somewhat constrained.

**Discussion:**

Surgical methods encompass endoscopic forehead lift, eyebrow lift techniques, and scalp excision‐based forehead reduction surgery, each with specific advantages and drawbacks. Thread lifting bridges the gap between surgical and non‐surgical modalities. Forehead areas often lack subcutaneous fat, requiring precise anatomical understanding for effective thread lifting. Cannula usage with partial tunneling ensures efficacy even in patients with strong adhesions. Presented cases showcase successful eyebrow lifting using cog threads, illustrating immediate and gradual post‐procedure changes.

**Conclusion:**

Eyebrow thread lifting demands periodic procedures, offering subtler improvements than surgery. Anatomical knowledge is crucial, and the technique presents discomfort. Effects last about 6 months, requiring re‐treatment as effects regress. Thread lifting, a middle ground between surgical and non‐surgical methods, can rejuvenate brows with less downtime.

## 1 INTRODUCTION

While every surgery is tailored to address the specific aging‐related changes in individual patients, many individuals considering blepharoplasty often exhibit some degree of eyebrow ptosis.^1^ Therefore, it is crucial for oculofacial plastic surgeons to have a thorough grasp of surgical anatomy and techniques for rejuvenating the brow and forehead.^2^, ^3^ Unlike the standardized technique commonly used for upper and lower blepharoplasty, there are various surgical approaches available to address concerns related to the eyebrow and forehead.

In recent times, there has been a surge in the popularity of forehead lifting procedures using threads, although a more precise term would be eyebrow lifting. As individuals age, the effects of gravity often lead to sagging of the forehead and eyebrows. This can cause the skin on the eyelids to droop, obscuring the upper eyelid line and creating a sensation of heaviness around the eyes. Conversely, raising the eyebrows can diminish the appearance of eye droopiness, improving the visual field and imparting a revitalized look. However, it is important to note that non‐surgical methods may not effectively address all instances of sagging skin between the eyebrows and eyelids, necessitating surgical intervention.^3^, ^4^, ^5^, ^6^, ^7^

Non‐surgical techniques for eyebrow lifting may involve the use of botulinum neurotoxin (BoNT) to weaken the muscles responsible for pulling the eyebrows downward. However, the results of BoNT treatment may not always be pronounced. Targeting specific muscles involved in brow depression, such as the orbital orbicularis oculi, procerus, corrugators, and depressor supercilli, can provide some elevation to the medial and lateral eyebrow area, but the effects are typically limited.

Another non‐surgical method involves using filler to support the orbicularis oculi muscle, thereby lifting the lateral part of the eyebrow. By strategically injecting high‐G' (elastic modulus) and high‐viscosity fillers into the pre‐periosteal area, a lifting effect can be achieved, providing resistance to both gravity and facial movement. However, this effect is often subtle.^8^ Surgical approaches include procedures such as endoscopic forehead lift, various eyebrow lift techniques (such as sub‐brow blepharoplasty and supra‐brow methods), and forehead reduction surgery involving scalp excision. Endoscopic forehead lift surgery may inadvertently result in the backward retreat of the hairline, leading to a secondary effect of widening the forehead. Conversely, scalp excision‐based forehead reduction surgery carries the risk of scarring near the hairline area.

Thread lifting serves as an intermediary between surgical and non‐surgical approaches, playing a crucial role in addressing eyebrow ptosis and forehead sagging.

## 2 ANATOMICAL CONSIDERATION

In forehead region, subcutaneous fat layers are often sparse, and the connective tissue beneath the skin tends to be tightly adhered. Therefore, a thorough understanding of the anatomy is crucial for optimizing the effectiveness of thread lifting procedures in the brow area. To ensure a smooth procedure, a cannula is used to partially create tunnels through the loose areolar tissue beneath the forehead muscles along the path of thread insertion, even in patients with strong tissue adhesions. Before initiating the procedure, it is important to evaluate the anticipated results and determine whether more aggressive cannula dissection is necessary to establish adequate procedural space, especially in areas with significant adhesions.

Typically, the entry point is along the lower margin of the eyebrow, and the extent of traction is determined based on the degree of eyebrow laxity in the patient. Inserting threads at the hairline can be uncomfortable as hair strands tend to intertwine. However, reverse insertion allows effective lifting by elevating the targeted lower tissues and securing the threads in firmer tissue. A frequently used type of polydioxanone cog thread, typically 6–8 cm in length, integrated with a cannula is preferred for this purpose.

**FIGURE 1:**
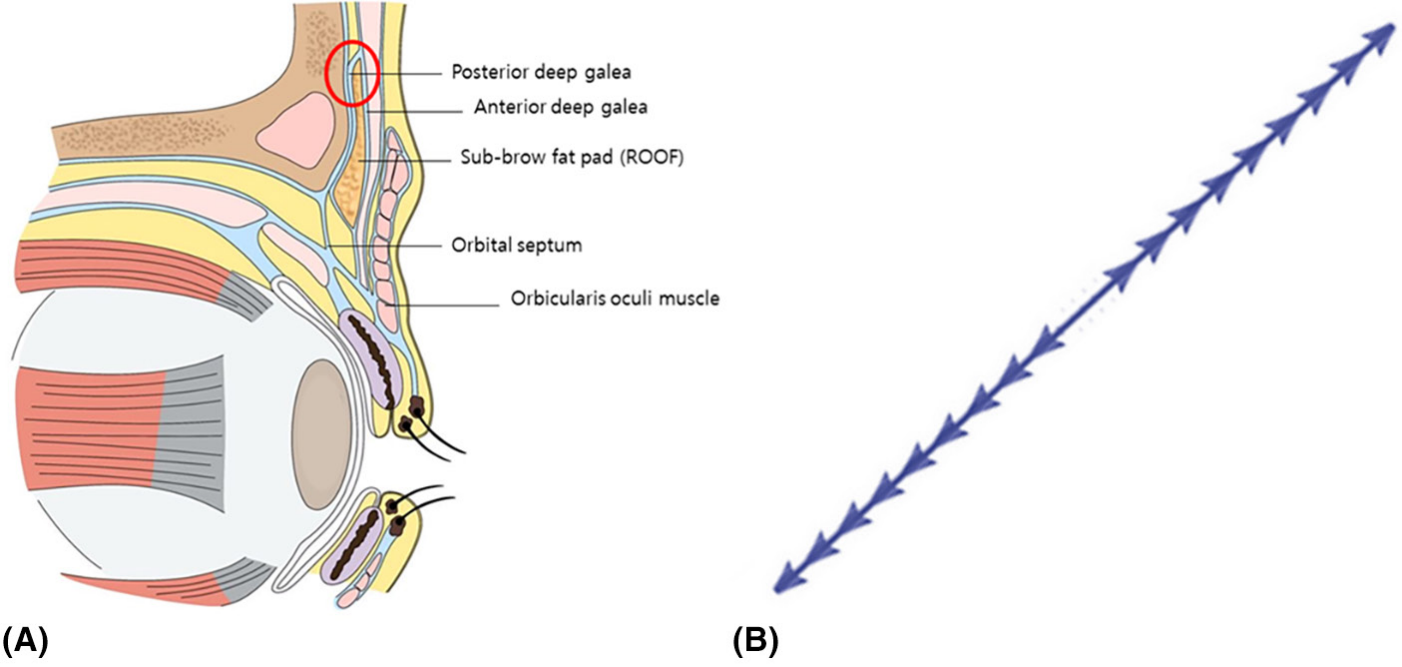
Typically, cog threads with a cannula are often used for eyebrow lifting. An 18G cannula is inserted and fixed in the orbital retaining ligament (red circled) after local anesthesia, passing beneath the forehead muscles into the subgalea‐frontalis space (A). Care is taken to avoid damaging nerves or vessels by ensuring the cannula follows the curved forehead and stays close to the subperiosteal layer. Special attention is needed when passing through the orbicularis retaining ligament to prevent a sensation of pulling on eyebrow tissues. The thread used are Secret line Illusion 7 cm long (Hyundai Meditech., Inc., Wonjusi, Republic of Korea) (B).

The entry point for the cog thread is locally anesthetized, and a needle puncture is made, following which an 18G cannula is used to pierce through the forehead muscles and advance below the muscles. Upon confirming the periosteum beneath the muscles, gently progressing the cannula in the supraperiosteal layer allow access to the subgaleal‐frontalis space composed solely of fibrous tissue (Figure 1).

However, if the cannula's direction does not stay in this exact plane past the contours of the forehead and continues upward, penetrating the muscle layer, it may risk damaging nerves or blood vessels. To prevent this, individuals with pronounced forehead contours should tent or curve the cannula in a convex configuration before the thread insertion, ensuring the cannula consistently remains close to the periosteum, guiding it carefully to avoid muscle penetration.

During this process, attention should be paid to the depth and position as the tip of the cannula passes through the orbicularis retaining ligament, situated approximately 2–3 mm above the superior orbital rim, acting as a barrier between the spaces of the forehead and orbit. This ligament prevents the eyebrow tissues from sagging. Passing the cog thread through this ligament might create a sensation of pulling and redraping the tissue, indicating a successful insertion. This technique demonstrates a major difference from surgery, as it allows lifting of the eyebrows without altering the hairline.

### 2.1 Clinical case 1

**FIGURE 2:**
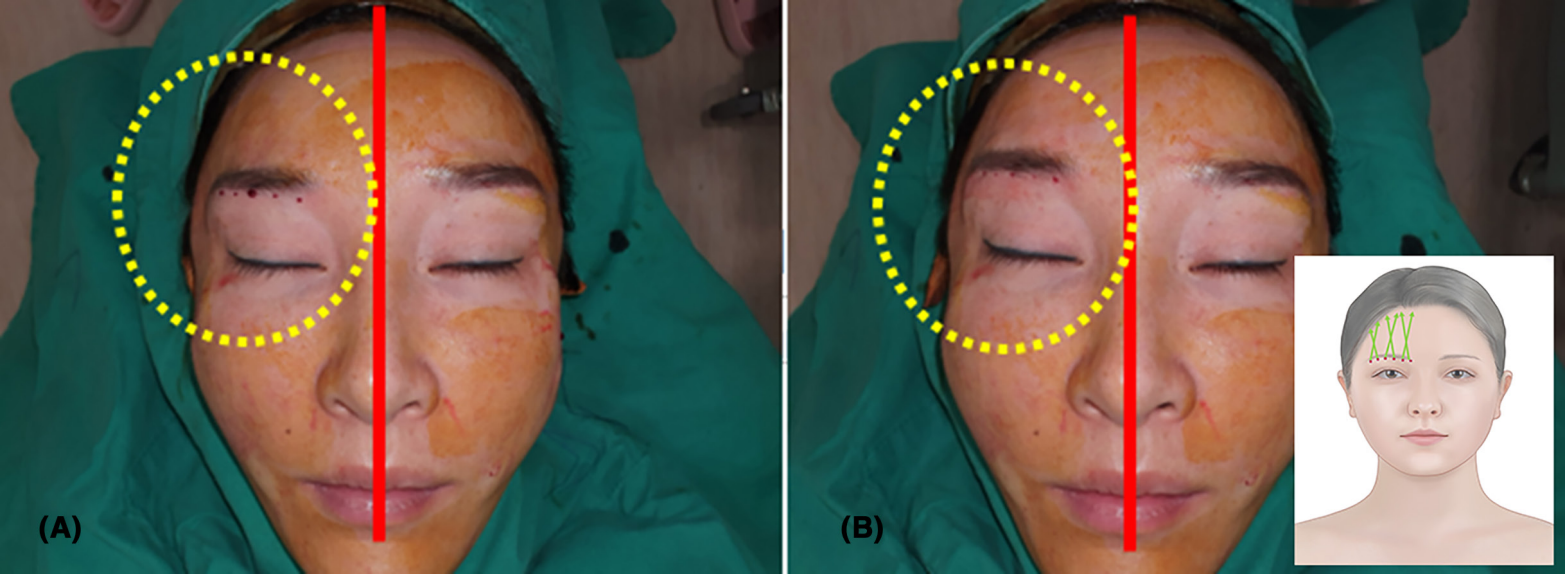
A 42‐year‐old woman underwent eyebrow lifting using 7 cm Secret line illusion 7 cm long (Hyundai Meditech., Inc., Wonjusi, Republic of Korea) on her right side. The entry point was below the right eyebrow, as seen in panel A with minor bleeding caused by an 18G needle. Employing a criss‐cross ‘X' pattern, six points were threaded to lift the eyebrow, evident in panel B, demonstrating a noticeable elevation of the right eyebrow compared to the untreated left side.

This patient is a 42‐year‐old female who visited the clinic with concerns of drooping eyebrows. The case involved using a 8 cm cog thread to lift only the right eyebrow. The entry point was chosen below the right eyebrow. In Figure 2A, only the puncture with an 18G needle is illustrated, resulting in minor bleeding to mark the entry point. Utilizing a criss‐cross pattern resembling an ‘X' shape, the thread was inserted at six points to lift the eyebrow immediately after (Figure 2B). By comparing the untreated left eyebrow to the right side where the lifting was performed, a noticeable elevation of the right eyebrow can be observed.

### 2.2 Clinical case 2

**FIGURE 3:**
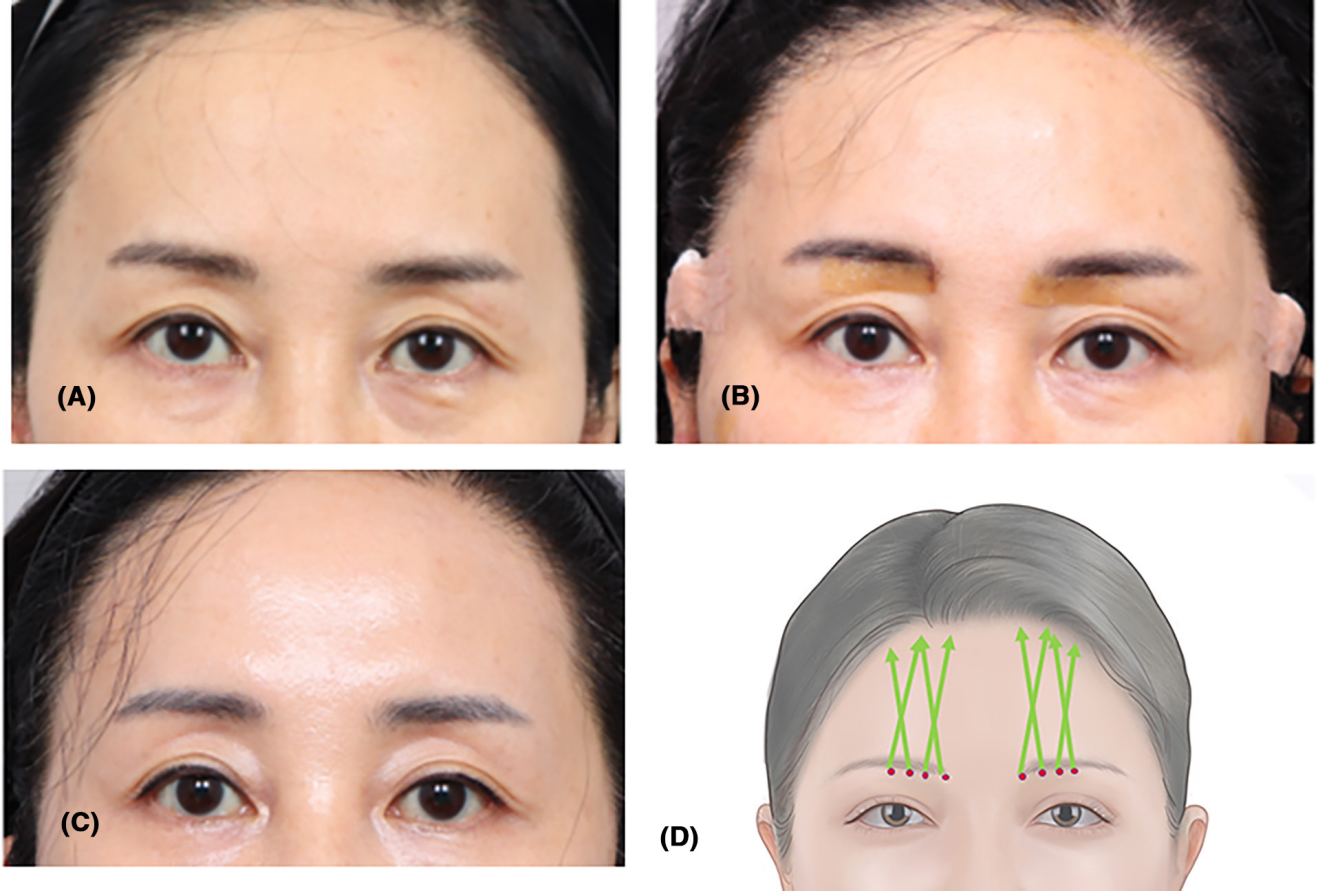
A 53‐year‐old patient received treatment targeting the front part of the eye area. The images show pre‐procedure, immediate post‐procedure, and one‐month follow‐up (A–C). This method suits those with minor eyebrow drooping and limited skin laxity, avoiding incisions due to excessive skin. It is ideal for patients with mobile eyebrows. It is better to perform the procedure without using tumescent solution when possible. The insertion, done slightly above the frontalis muscle layer after a small puncture, creates space for easy thread placement by gently pinching the tissue (D). The thread used are Secret line Illusion 7 cm long (Hyundai Meditech., Inc., Wonjusi, Republic of Korea).

A 53‐year‐old woman primarily sought to lift the front part of her eye area, which was the focal point of the lifting procedure. The accompanying images depict the condition before the procedure, immediately afterward, and one‐month post‐procedure (Figure 3A–C). This technique is well‐suited for patients with narrower spaces between the eyebrows and eyelids, experiencing drooping eyebrows and moderate skin laxity. It is particularly suitable for individuals without an excess of redundant skin that requires incisions and is better suited for those with mobile eyebrows (Figure 3D).

Some practitioners prefer to use a tumescent solution, while others do not. Although the tumescent solution offers benefits such as mild tissue dissection and pain reduction, there is a potential concern for prolonged swelling, and impediment of the ability of the cogs to attach to its targeted tissue. Consequently, preference is often given to conducting the procedure without tumescence whenever possible.

Insertion is done slightly above the frontalis muscle layer following a minor puncture. By gently pinching the tissue, a space is generated, giving less resistance to insert the precannulated thread.

### 2.3 Clinical case 3

**FIGURE 4:**
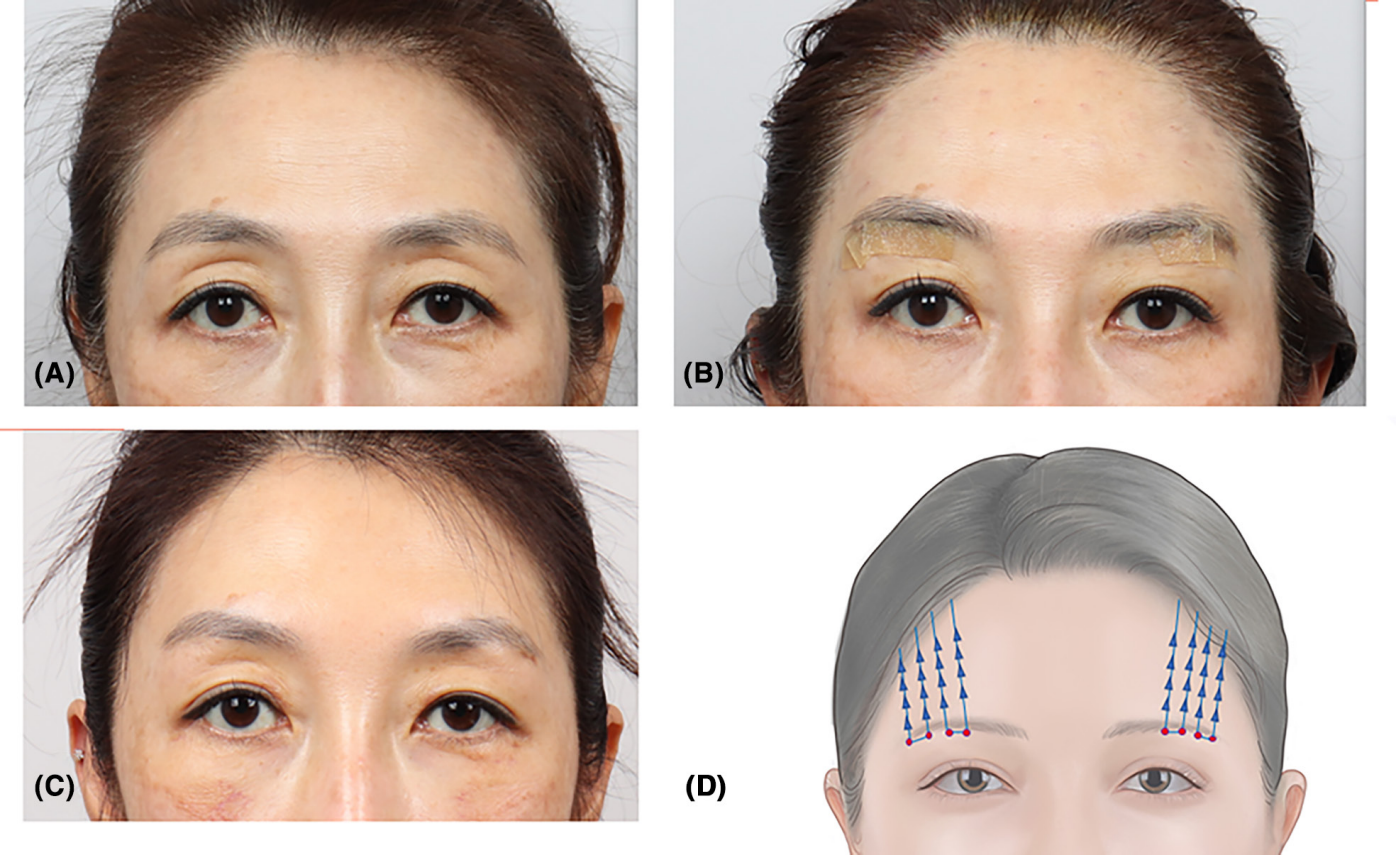
A 35‐year‐old female displays lateral hooding, characterized by the drooping of the outer part of the eyebrows that cover the upper eyelid fold. Enhancement in the appearance was accomplished using eyebrow thread lifting techniques. The images illustrate the condition before the treatment (A), directly after the procedure (B), and 3 months post‐treatment (C). Secret line Double S Miracthreads (Hyundai Meditech, Wonjusi, Korea) were employed, inserted in a U‐shaped arrangement, employing two threads on each side (D).

This 35‐year‐old female patient presented with lateral eyelid hooding, where the outer part of the eyebrows drooped, covering the upper eyelid fold. Improvement in her appearance was achieved through eyebrow thread lifting, using Secrete threads (Hyundai Meditech, Wonjusi, Korea) inserted in a U‐shaped pattern with two threads per side. The images depict the condition before the procedure (Figure 4A), immediately after the procedure (Figure 4B), and 3 months following the procedure (Figure 4C). Secrete threads (Hyundai Meditech, Wonjusi, Korea) were deployed in a U‐shaped pattern, with two threads per side (Figure 4D).

Video S1 provides a demonstration of the thread insertion technique.

## 3 DISCUSSION

Various methods for brow lifting are introduced, including one that utilizes long threads inserted through cannulas, maneuvered, and then pulled back to shift the points.^9^ However, this method presents challenges such as the difficulty of re‐inserting the extracted, particularly in outpatient settings under local anesthesia. As a result, consideration is given to utilizing a more manageable floating‐type cannula thread for eyebrow lifting.

Eyebrow thread lifting requires periodic repeat procedures and generally yields less dramatic improvements compared to surgery. Nonetheless, it can elevate drooping eyebrows, offering a more rejuvenated appearance, and revealing previously concealed eyelid creases. It is important to note that discomfort in the forehead area might be experienced, thus requiring thorough informed consent.

The effects of eyebrow thread lifting typically last for approximately 6 months. However, over time, they gradually diminish, returning towards the original position. Re‐treatment becomes necessary as the effects gradually wane rather than maintaining the same level for a full six‐month duration. Unlike lower face thread lifting, eyebrow thread lifting frequently leads to increased discomfort or pain. Nevertheless, this discomfort typically diminishes considerably within the first 2–3 days. Considering additional methods such as high‐intensity focused ultrasound is recommended to sustain the results.

The eyebrow complex holds significant aesthetic value in the upper face, and various techniques exist for brow lifting, each with distinct advantages and limitations. Surgeons should be proficient in multiple methods to address diverse patient needs. Surgical approaches such as transblepharoplasty and direct techniques effectively address brow ptosis but may not adequately target forehead wrinkles. Direct brow‐lift methods are particularly beneficial for patients with facial paralysis requiring significant elevation of a drooping brow; these individuals are often more accepting of resulting scars.^4^

Saltz and Lolofie^10^ advocate for endoscopic brow‐lift surgery as the gold standard for forehead rejuvenation. They highlight its superior capacity for safe tissue release and the mitigation of complication risks, emphasizing personalized aesthetic outcomes based on specific forehead characteristics, such as flatness, non‐receding hairlines, and minimal redundant skin, and individual patient factors such as gender, ethnicity, and facial aging.

Patrocinio et al.^11^ explore the transpalpebral eyebrow lift as surgical approach for mild‐to‐moderate brow ptosis and post‐upper blepharoplasty brow descent. The authors compare its advantages, including minimal incisions and direct visualization of anatomical structures, to the endoscopic brow‐lift. The study outlines surgical techniques, upper face rejuvenation steps, and complications management, cautioning against its use in severe brow ptosis and marked forehead aging.

Fattahi^12^ examines the pretrichial brow lift, a form of open brow lift surgery, highlighting its advantages over alternative methods. He emphasizes its superiority over alternative methods, noting its capacity to shorten an elongated forehead, reposition the hairline anteriorly, and its relative simplicity compared to other procedures.

In contrast, Sahan et al.^13^ investigated the efficacy of a thread lift technique for brow lifting in 50 female patients, aiming to counteract brow descent and assess its outcomes. Using polydioxanone threads, the minimally invasive approach yielded significant improvements in brow position, with 48% of patients reporting very much improved results based on the global aesthetic improvement scale. Complications, including minor issues like ecchymosis and edema, were reported in 18% of patients but resolved within 10 days. Patient satisfaction was high, with 60% feeling extremely satisfied with the procedure. The study suggests that the described technique offers prolonged results with minimal downtime, addressing a common concern in thread lift procedures. Despite limitations such as its retrospective nature and small sample size, the findings underscore the potential for safe, effective, and long‐lasting brow rejuvenation with the thread lift technique.

In conclusion, thread lifting emerges as a promising non‐surgical alternative approach for brow lifting. Positioned between surgical and non‐surgical methods, it requires skill and expertise but offers a less invasive option with potential for effective brow rejuvenation. This middle ground may appeal to individuals seeking subtle enhancements with minimal downtime.

## AUTHOR CONTRIBUTIONS

All authors have reviewed and approved the article for submission. Conceptualization, Kyu‐Ho Yi, Soo Yeon Park. Writing—original draft preparation, Kyu‐Ho Yi, Soo Yeon Park, Jovian Wan, Thanawan Sirisuk. Writing—review & editing, Kyu‐Ho Yi, Soo Yeon Park, Jovian Wan, Thanya Techapichetvanich. Visualization, Kyu‐Ho Yi, Soo Yeon Park, Ti Jo Tsay. Supervision, Kyu‐Ho Yi, Soo Yeon Park, Soo‐Bin Kim, Jovian Wan, Ti Jo Tsay.

## FUNDING INFORMATION

There is no financial disclosure to report.

## CONFLICT OF INTEREST STATEMENT

I acknowledge that I have considered the conflict of interest statement included in the “Author Guidelines.” I hereby certify that, to the best of my knowledge, that no aspect of my current personal or professional situation might reasonably be expected to significantly affect my views on the subject I am presenting.

## ETHICS STATEMENT

Authors declare human ethics approval was not needed for this study.

## Supporting information


Video S1:

## Data Availability

Data sharing is not applicable to this article as no new data were created or analyzed in this study.
